# Arthropathy-related pain in a patient with congenital impairment of pain sensation due to hereditary sensory and autonomic neuropathy type II with a rare mutation in the *WNK1/HSN2* gene: a case report

**DOI:** 10.1186/s12883-016-0727-8

**Published:** 2016-10-21

**Authors:** Keiko Yamada, Junhui Yuan, Tomoo Mano, Hiroshi Takashima, Masahiko Shibata

**Affiliations:** 1 Center for Pain Management, Osaka University Hospital, 2-15 Yamadaoka, Suita-shi, Osaka, 565-0871 Japan; 2 Public Health, Department of Social Medicine, Osaka University Graduate School of Medicine, 2-2 Yamadaoka, Suita-shi, Osaka, 565-0871 Japan; 3 Department of Neurology and Geriatrics, Kagoshima University Graduate School of Medical and Dental Sciences, 8-35-1 Sakuragaoka, Kagoshima, 890-8520 Japan; 4 Department of Neuromodulation, Osaka University Graduate School of Medicine, 2-2 Yamadaoka, Suita-shi, Osaka, 565-0871 Japan; 5 Department of Pain Medicine, Osaka University Graduate School of Medicine, 2-2 Yamadaoka, Suita-shi, Osaka, 565-0871 Japan

**Keywords:** Hereditary sensory and autonomic neuropathies, Arthropathy, Demyelinating diseases, Acetaminophen, Case report

## Abstract

**Background:**

Hereditary sensory and autonomic neuropathy (HSAN) type II with *WNK1/HSN2* gene mutation is a rare disease characterized by early-onset demyelination sensory loss and skin ulceration. To the best of our knowledge, no cases of an autonomic disorder have been reported clearly in a patient with *WNK/HSN2* gene mutation and only one case of a Japanese patient with the *WNK/HSN2* gene mutation of HSAN type II was previously reported.

**Case presentation:**

Here we describe a 54-year-old woman who had an early childhood onset of insensitivity to pain; superficial, vibration, and proprioception sensation disturbances; and several symptoms of autonomic failure (e.g., orthostatic hypotension, fluctuation in body temperature, and lack of urge to defecate). Genetic analyses revealed compound homozygous mutations in the *WNK1/HSN2* gene (c.3237_3238insT; p.Asp1080fsX1). The patient demonstrated sensory loss in the “stocking and glove distribution” but could perceive visceral pain, such as menstrual or gastroenteritis pain. She experienced frequent fainting episodes. She had undergone exenteration of the left metatarsal because of metatarsal osteomyelitis at 18 years. Sural nerve biopsy revealed a severe loss of myelinated and unmyelinated nerves. She complained of severe pain in multiple joints, even on having pain impairment. Although non-steroidal anti-inflammatory drugs are generally more effective than acetaminophen for arthritis, in our case, they were ineffective and acetaminophen (2400 mg/day) adequately controlled her pain and improved quality of life. Over 3 months, the numerical rating scale, pain interference scale of the Brief Pain Inventory, and the Pain Catastrophizing Scale decreased from 6/10 to 3/10, from 52/70 to 20/70, and from 22/52 to 3/52 points, respectively.

**Conclusions:**

This is the second reported case of a Japanese patient with *WNK/HSN2* gene mutation of HSAN type II and the first reported case of an autonomic disorder in a patient with the *WNK/HSN2* gene mutation. Acetaminophen adequately controlled arthropathy related pain in a patient with congenital impairment of pain sensation.

## Background

Hereditary sensory and autonomic neuropathies (HSANs) are clinical and genetic disorders of the peripheral nerve [1]. HSANs are linked to 12 genes and have been classified into types I–V on the basis of age at onset, mode of inheritance, and predominant clinical symptoms [1]. Patients with HSAN type II present with loss of pain, temperature, and touch and mutations in the hands and feet [1]. Autonomic disorder is not a dominant feature of HSAN type II, although this disease is called “hereditary sensory and *autonomic* neuropathy” [1]. HSAN type II, with mutation in the nervous system-specific HSN2 exon of the with-no-lysine(K)-1 (*WNK1*) gene (*HSN2/WNK1*), is a very rare autosomal recessive disease. A few cases of *WNK1/HSN2* have been reported among the following ethnic groups: French–Canadian families (c.594delA, c.918_919insA, c.943c > T) [2, 3], a Lebanese family (c.947delC) [4], and two British families (c.60_61delTG + c.1168_1171delACAG and c.1168_1171delACAG + c.1168_1171delACAG) [5] and in Austrian (c.550C > T) [6], Italian (c.255delC, c.1089_1090insT) [6], Belgian (c.1064_1065delTC) [6], Polish (c.539_540delAG, c.2897_2898delAG) [7], Korean (c.1134_1135insT, c.217C > T) [8], and Japanese (c.1134_1135insT) [9] ethnicities. With the exception of dry hands in a Korean case, autonomic complications have not been reported in patients with HSAN type II with *HSN/WNK1* mutation [8].

Charcot arthropathy includes deforming and destructive process in joints and is one of the complications of neurosensory disorders [10]. The lack of protective sensation in patients with sensory neuropathies could cause delayed identification of bone injuries by overload [10]. Because of the lack of sensation experienced by patients with Charcot arthropathy, they are not expected to experience much pain despite severe deformation. However, a previous study reported that among 55 patients with Charcot arthropathy, more than 75 % complained of pain in the foot at the final stage of deformation, although all patients had clinical loss of sensation [11]. The reasons for this remain unclear.

## Case presentation

A 54-year-old woman presented with loss of touch, temperature, position, and vibration sense and taste disorder, and she was insensitive to pain at the body surface since infancy. She also suffered from an autonomic disorder, with symptoms, such as orthostatic hypotension, fluctuation in body temperature, and lack of urge to defecate.

**Fig. 1:**
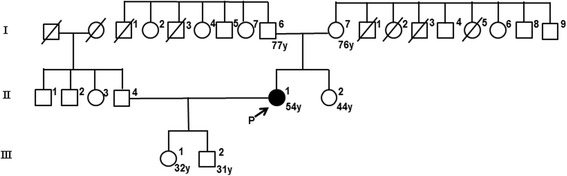
The family tree of the patient

She had normal mental growth and development, although she did not walk until 18 months of age. Her family history was unremarkable; she had a healthy younger sister and her parents were not related (Fig. 1). She had repeated injuries during childhood because of insensitivity to pain, and she was diagnosed with Freiberg disease at 6 years. She experienced frequent fainting episodes and was diagnosed with an autonomic nervous system imbalance at 21 years. Even in cold temperatures, she perspired on her back and reported not needing a snowsuit. She did not have high blood pressure.

After repeated jumping from a squatting position in gym class at 14 years, the sole of her left foot developed severe blisters, and the wounds transitioned into metatarsal osteomyelitis after a few weeks. As a result of the refractory osteomyelitis, a left metatarsal was replaced by an autogenous bone graft when she was 18 years. It took approximately 10 years to recover fully from the wounds.

She had migraine since childhood until she was 20 years and took a painkiller (an antipyrine medication) daily during that time. Although she could feel a toothache/headache, she lacked sensation on the surface of her face, except around the jaw. She showed a “stocking and glove distribution”, and the detection range of sensation varied along a gradient from the periphery to center. She could perceive visceral pain, such as menstrual or gastroenteritis pain, which she controlled with an antipyrine medication. Since 42 years of age, she started using a cane for walking outside.

Although she had known that she was different from others concerning pain perception since childhood, she and her parents had never consulted a doctor regarding the impaired pain sensation until 48 years of age.

**Fig. 2:**
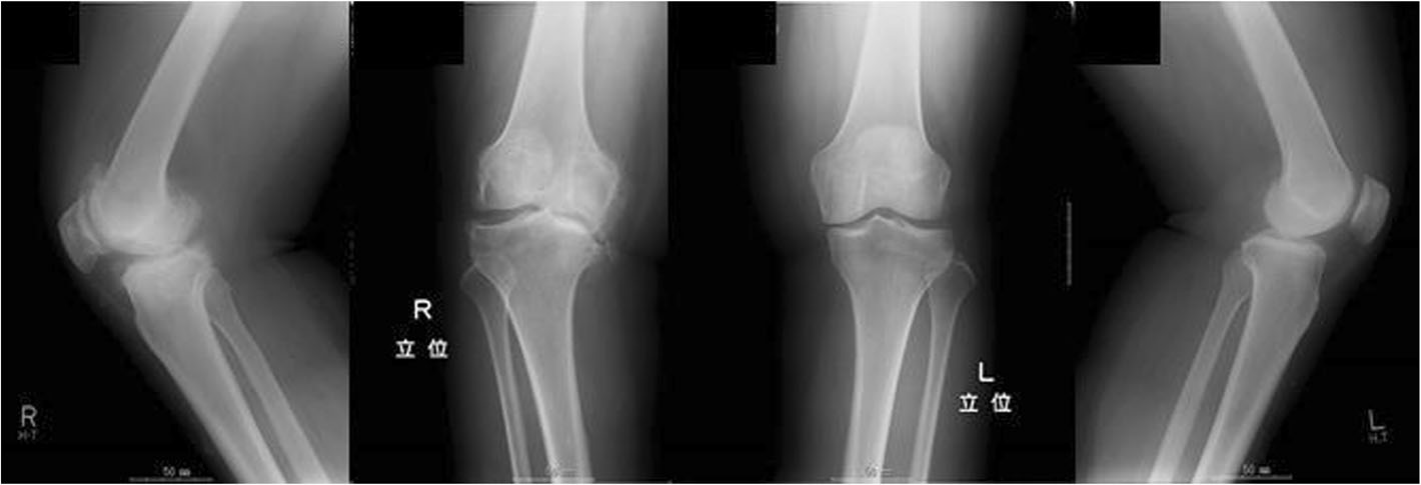
X-ray photograms of the legs

At 48 years of age, the patient developed sudden severe pain and swelling in her right joint, and her previous physician diagnosed Charcot arthropathy (X-ray photograms are shown in Fig. 2). At 50 years of age, she was confined to a wheelchair to avoid putting weight on her joints. The pain gradually affected multiple joints over subsequent years. Non-steroidal anti-inflammatory drugs (NSAIDs) improved her joint pain to a limited degree. At 54 years of age, her joint pain became intolerable. NSAIDs were not effective enough for her to resume activities of daily living. Mexiletine hydrochloride treatment was not effective. She experienced side effects of pregabalin and duloxetine. Subsequently, her physician referred her to our multidisciplinary center for pain management. She was diagnosed with HSAN type II using molecular genetic analysis and was prescribed acetaminophen (2400 mg/day), which controlled her pain very well and improved her quality of life.

During our follow-up, she reported: “one day severe ‘electric-shock-like or piercing pain’ occurred, and which made me suffer few times per hour and keeping for months after this episode, and which was naturally decreased.”

### Pain-related assessment

Brief Pain Inventory (BPI), including numerical rating scale (NRS) [12, 13], was used to assess pain intensity and interference. Pain-related psychosocial factors were quantitated using the Hospital Anxiety and Depression scale (HADS) [14] and Pain Catastrophizing Scale (PCS) [15]. NRS and pain interference scale of BPI decreased from 6/10 to 3/10 and from 52/70 to 20/70 points, respectively, over 3 months. PCS scores also decreased from 22/52 to 3/52 points. At baseline, HADS indicated normal mental state; both the anxiety and depressive scales of HADS were relatively low (both 5/21 points) despite severe pain. After 3 months, the anxiety and depressive scales of HADS decreased from 5/21 to 3/21 points and from 5/21 to 4/21 points, respectively.

### Neurological examination

A neurological examination revealed normal mental status, speech, and comprehension and intact cranial nerve-innervated muscles. Manual muscle testing revealed moderate weakness in the distal parts of the extremities and mild weakness in the proximal parts. Grip strength was significantly reduced (right, 4.0 kg; left, 6.0 kg). Deep tendon reflexes were absent. Plantar responses were flexor on both sides. The sensory examination revealed that the tactile and pinprick sensations were moderately decreased in the face and trunk and severely diminished in the upper and lower distal extremities. Sensations were diminished in a stocking and glove pattern. The vibration sense was reduced till the knees and absent in the ankles. The joint position sense was absent at the hallux. There was no apparent laterality of the sensory disturbance. Pseudoathetosis was observed in the upper limbs, and Romberg’s test was not positive in the seated position. With the exception of abnormal sweating, thermoregulatory failure, and lack of urge to defecate, there were no signs of autonomic dysfunction, such as pupillary responses, dry eyes, or dry mouth. Her blood pressure was 138/85 bpm in the supine position and 126/72 bpm while standing. The head-up tilt test did not reveal any orthostatic intolerance. On electrocardiogram, the coefficient of variation of the R-R intervals (CVR-R) measured at rest was normal. The early and delayed heart-to-mediastinum (H/M) ratio was not decreased on 123I-MIBG myocardial scintigraphy (early: 3.14, delay: 2.97).

### Nerve conduction and electromyographic evaluation

The nerve conduction study revealed normal compound muscle action potentials (CMAPs) values, except for a slightly reduced motor nerve conduction velocity of 50.0 m/s, 41.0 m/s, and 39.0 m/s in the median, peroneal, and tibial nerves, respectively. The velocity was in the normal range in the ulnar nerve (>50.5 m/s) and in the tibial and peroneal nerves (>48.0 m/s). The sensory nerve action potentials of the median, ulnar, and sural nerves were not evoked. The electromyography revealed a reduction in recruitment in the distal muscles of the upper and lower limbs. The patient was diagnosed with pure sensory neuropathy.

### Pathologic examination

**Fig. 3:**
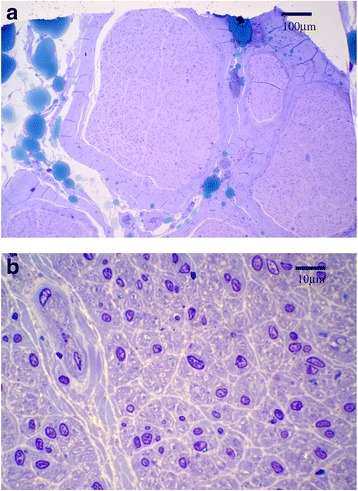
Sural nerve biopsy viewed under light microscopy of the epon section with toluidine blue staining. Light microscopy findings (**a**) Low-power image, **b** High-power image

**Fig. 4:**
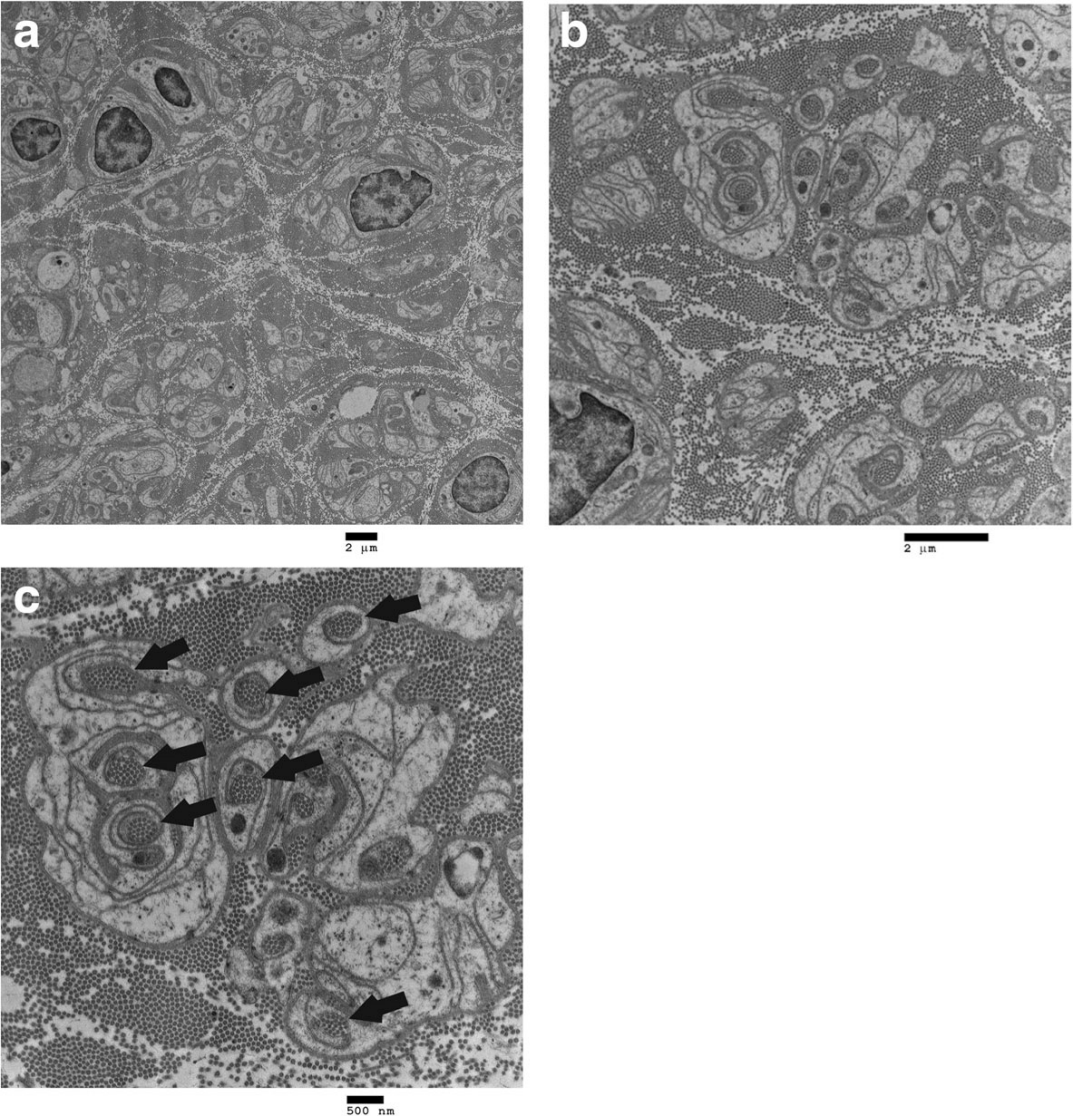
Sural nerve biopsy viewed under electron microscopy. Electron microscopy findings (**a**) Low-power image, **b** High-power image. Myelinated nerve completely disappeared, and the density of unmyelinated nerves (7620 per mm^2^) significantly decreased. **c** Collagen pockets are indicated by the arrows

Sural nerve biopsy revealed a severe loss of myelinated and unmyelinated nerve which were observed by light microscopy of the epon section with toluidine blue staining (Fig. 3a, b) and electron microscopy (Fig. 4a, b). Collagen pockets, which were indicative of unmyelinated nerve loss, were observed by electron microscopy (indicated by the arrows in Fig. 4c). Myelinated nerve tissue was completely lost. The density of unmyelinated nerve tissue (7620 per mm^2^), calculated by electron microscopy, was significantly decreased. Skin biopsy was not performed.

### Molecular genetic analysis

Mutation screening was conducted as previously described [16]. Target sequencing with 16 HSAN disease-related genes were conducted using Illumina MiSeq (Illumina Inc., San Diego, CA, USA). The *WNK1* mutation observed in this patient was validated by Sanger sequencing. A homozygous frame shift mutation was identified in the *WNK1/HSN2* gene c.3237_3238insT (p.Asp1080fsX1; ENST00000537687), which was previously reported as c.1134_1135insT (p.Asp379fsX1; ENST00000574564) [8, 9]. This mutation is absent in 1000 Genomes, ExAC, or HGVD, which comprises exome sequencing of 1208 Japanese individuals (http://www.genome.med.kyoto-u.ac.jp/SnpDB/).

## Conclusion

We would like to highlight three important points from this case. First, this is the second case of *WNK/HSN2* gene mutation of HSAN type II reported in a Japanese patient. Second, this is the first reported case of an autonomic disorder in a patient with the *WNK/HSN2* gene mutation. Third, the patient’s arthropathy-related pain, despite congenital impairment of pain sensation, was a noteworthy symptom.

The current patient is the second reported case of a patient with *WNK1/HSN2* gene mutation in Japan, both of which resulted from the same homozygous mutation, c.3237_3238insT [9]. *WNK1/HSN2* with homozygous 1134–1135 ins T mutation was reported in a Japanese patient by Takagi et al. [9], and the same mutation was reported in Korea by Cho et al. [8].

Although an autonomic disorder has never been reported in HSAN type II with the WNK1/HSN2 gene mutation, our patient presented with several symptoms of autonomic disturbances. In particular, the lack of urge to defecate through decreased parasympathetic pelvic nerve activity resulted in serious disruptions in her activities of daily living. Although there have been no reports of hyperhidrosis in patients with HSAN type II, fluctuation in body temperature is one of the most common symptoms of autonomic failure. In the presence of partial hyperhidrosis, there may be peripheral autonomic dysfunction. WNK influences transient receptor potential vanilloid 4 (TRPV4) channel function, which controls osmoregulation at the cellular level and regulates water balance. Therefore, there is an association between WNK1 and severe hypertension as reported previously [17]. However, our patient did not present with hypertension. Although our examination did not reveal autonomic dysfunction, we think that the possibility of autonomic failure cannot be excluded. This is the first reported case of an autonomic disorder in a patient with the *WNK/HSN2* gene mutation, and HSAN type II should be carefully considered because symptoms of autonomic dysfunction appeared in our patient.

Recurrent skin ulcers on the tips of the fingers and toes were previously reported [1, 8, 9]. Our patient had a difficult in recovering from injury and an amputated metatarsal due to osteomyelitis, but she did not develop skin ulcers. Except the autonomic disorder, her neurological examination had almost the same clinical features as those of previous patients with HSAN type II with *WNK1/HSN2* gene mutations.

NSAIDs are generally more effective than acetaminophen for arthritis with inflammation. However, in our patient, acetaminophen was more effective than NSAIDs for alleviating multiple joint pain. Theoretically, her arthropathy pain was conducted by unmyelinated nerve fibers (C fibers) and was possibly modified by central pain because acetaminophen was very effective for controlling her pain, although she had a reduction in unmyelinated nerve tissue. The antinociceptive mechanism of acetaminophen is still unclear, but the theory of multiple pathways is supportive. Although acetaminophen inhibits cyclooxygenase (COX), anti-inflammatory effect of acetaminophen is weak. Acetaminophen is lipid-soluble, passes through the blood–brain barrier, and has an effect on the central nerve system [18]. Acetaminophen activates 5-hydroxytryptamine 3 receptors in the serotonergic pathways, which are part of the descending pain system, with resulting pain relief [19]. The gamma-aminobutyric acid receptor is associated with acetaminophen [20]. Cannabinoid is one of the key factors of acetaminophen-induced antinociception [21]. Moreover, TRPV1 has an important role in antinociception induced by acetaminophen in the brain [22].

The patient felt non-specific ‘electric shock-like or piercing’ pain suddenly, but she was innately unable to feel sharp pain because of complete demyelination. Her non-specific pain might have been phantom pain, derived from the central nervous system because it was correlated with her strong emotional episode. When she complained about non-specific pain, her father was diagnosed of cancer and reported a pins-and-needles sensation in his fingers due to peripheral nerve disorder, which was caused by anti-cancer chemotherapy. He had complained about the pins-and-needles sensation every day, and she was told that she had empathized with and imagined his pain. We suspect that her non-specific pain was central pain from this emotional episode relating to her father. A previous study reported that empathy for pain may effectively activate pain neural circuits in an individual observing another person’s pain [23]. Consistent with our findings, Danzier et al. reported a 32-year-old woman with HSAN type V who experienced tension-type headaches shortly after the sudden loss of her brother, despite complete absence of physical pain [24].

## Abbreviations

BPI: Brief Pain Inventory
CMAPs: Compound muscle action potentials
HADS: Hospital Anxiety and Depression scale
HSAN: Hereditary sensory and autonomic neuropathy
NRS: Numerical rating scale
NSAIDs: Non-steroidal anti-inflammatory drugs
PCS: Pain Catastrophizing Scale
TRPV4: Transient receptor potential vanilloid 4
WNK1: With-no-lysine(K)-1
